# Assessing Perceptions of and Responses to Multiple Health Risks Among the Southern Poor

**Published:** 2010-12-15

**Authors:** Shelly R. Hovick, C. Ashani Johnson-Turbes, Doryn D. Chervin, Vicki S. Freimuth

**Affiliations:** University of Texas MD Anderson Cancer Center. At the time of this study, Dr Hovick was affiliated with the University of Georgia in Athens, Georgia; ICF Macro, Atlanta, Georgia; ICF Macro, Atlanta, Georgia; University of Georgia, Athens, Georgia

## Abstract

**Introduction:**

We explored perceptions of and responses to multiple health risks among people living in poverty in the southern United States.

**Methods:**

We conducted 12 focus groups and interviewed 66 focus group participants in 3 southern US cities (Birmingham, Alabama; Jackson, Mississippi; and Columbia, South Carolina). Thematic analysis was used to identify major themes.

**Results:**

Study participants worried most about chronic health conditions and the costs to treat those conditions. Feelings of threat were influenced by family health history and race. Barriers to health-protective behaviors included time, work, family, apathy, and low response efficacy. Physical activity and checking blood pressure were the health-protective behaviors in which participants most often engaged.

**Conclusion:**

Our results will be useful for the development of interventions that target the southern poor. Intervention messages should address the barriers that poor people face when attempting to engage in health-protective behaviors and should help strengthen people's confidence in their ability to change their behaviors.

## Introduction

Many studies of health knowledge and protective behaviors reveal disparities that are based on socioeconomic status (1,2). The poor have less knowledge about health risks (3), have a higher prevalence of risky behaviors (4), and are less likely to engage in protective behaviors than people at higher income levels (5-7). To address these disparities, developing an understanding of the cultural and social factors that affect the health behaviors and decisions made by people in poverty is essential.

Health disparities across income strata are well documented. The risk of premature death for people in poverty is double that of people with higher incomes (8), and they are at increased risk for several types of cancer, cardiovascular disease, and diabetes (9-12). People who are poor also use preventive care less, spend more time obtaining care, and are less informed about the benefits of disease screening (13). Having a low income has also been associated with having a poor diet, low physical activity, and an above-average prevalence of drinking (14).

More than 27% of people in Jackson, Mississippi, live below the federal poverty level, followed by 24% in Birmingham, Alabama, and 21% in Columbia, South Carolina (15-17). All 3 cities are in states with poverty rates above the national mean (18). In 2007, Mississippi, Alabama, and South Carolina ranked 1st, 6th, and 12th nationally in poverty, respectively (19). Data compiled by the Kaiser Family Foundation (20) show that these states have high rates of chronic disease. Death rates from cancer in these states are among the top 30% in the United States (21). Mississippi has the highest death rate from heart disease in the country, and Alabama is a close third (22). These states also have higher rates of smoking (23) and obesity (24), and higher prevalence of diabetes (25) than other US states. Additionally, 19% of people living in the South were uninsured compared with an average of 16% nationally (26). Because this subgroup of the population experiences health disparities, our objective was to better understand how people in this subgroup perceived multiple health risks and coped with them on limited resources.

The following questions guided our investigation: 1) What health conditions are participants most concerned or worried about?, 2) What factors contribute to participants' feelings of threat (severity and susceptibility)?, 3) What factors influence participants' decision making about health behaviors?, and 4) What health-protective behaviors do participants adopt?

## Methods

Unlike most studies that focus on 1 health risk at a time, we examined multiple health risks that cut across several areas, including chronic conditions, accidents, sexually transmitted diseases, and alcohol and drug use. We sought to understand what factors contribute to low-income people's feelings of threat and what issues shape their decision making about engaging in health-protective behaviors. Therefore, we concentrated on 3 critical constructs found in most behavior change models (27): 1) threat, 2) barriers, and 3) response efficacy.

The moderator's guide and interview instruments explored these 3 constructs and their influence on people's health-protective behaviors. To assess threat, we examined how susceptible participants felt they were to a range of health risks and how severe they believed each would be if they experienced them. To better understand the factors influencing decisions to engage in health-protective behaviors or not, we examined perceived barriers (particularly those that may be intensified by participants' income status) and response efficacy. Response efficacy refers to whether participants believe a recommended response will avert the threat (27).

We conducted 12 focus groups from August through December of 2006 in 3 southern US cities, which were followed by individual interviews with some of the focus group participants, to understand how poor people perceive and respond to various health risks. Participants (n = 71) were from Birmingham, Alabama; Jackson, Mississippi; and Columbia, South Carolina.

Focus groups are a flexible tool for exploring respondent awareness, behavior, concerns, beliefs, experiences, motivation, operating practices, and plans (28) and are well-suited for research with minority and vulnerable populations (29).

After we obtained approval from ICF Macro's institutional review board (given full authority by the University of Georgia to review and approve), we contracted with focus group facilities in each city to recruit participants and host the focus groups. The professional facilities used existing databases of potential research participants for recruitment. Potential participants were screened for eligibility on the basis of age (30-50 y), race (African American or white), and annual household income (≤$25,000). The income cutoff was slightly higher than the federal poverty level to account for the working or nearly poor. People who had participated in a focus group within the past year were not eligible. Four to six people participated in each focus group, and groups were separated by race and sex. Because of financial constraints, we did not sample higher income groups or stratify by geographic locale to account for neighborhood level influences. Additionally, because of time constraints, we did not collect more specific demographic information from participants. However, focus group participants were split nearly equally in terms of race and sex.

Focus groups consisted of a 90-minute, large-group audiotaped discussion, which was followed by a 30-minute individual interview. Two members of the research team (matching the race of the participants) moderated all focus groups. Moderators solicited participant concerns and attitudes about multiple health risks, feelings of efficacy, and barriers to health-protective behaviors. Moderators also gave participants a health scenario in which the participants were asked to imagine a sudden and potentially serious symptom and describe their process for gathering information and responding to it. In audiotaped interviews conducted by the research team, interview participants were shown a picture of 5 health-protective behaviors (getting an influenza vaccination, regularly checking blood pressure, doing physical activity, wearing a seatbelt, and preparing for a natural disaster) and were asked to describe their engagement in the behaviors. They were also asked to provide reasons for, barriers to, and motivators for engagement, as well as information-seeking behaviors. Interviews were used to obtain accurate indicators of actual behaviors that were not subject to potential group pressure.

All focus group audiotapes were transcribed verbatim by a professional transcriptionist. The research team conducted a thematic analysis to identify common themes and patterns among the focus groups. Two members of the research team developed an initial codebook to identify themes and patterns. The 2-person analysis team compared codes, themes, and patterns found in the data until no new codes (or code categories) emerged (30). Once saturation of codes was reached, we finalized the codebook to code all transcripts and identify focus group themes. A member of the research team applied the codes to the remaining focus group transcripts by using ATLAS.ti (Scientific Software Development GmbH, Berlin, Germany). After coding, the analysis team met frequently to discuss the coded findings and ensure ongoing agreement on code definitions and application to the data. The team concluded that the same or similar themes and patterns emerged consistently across focus groups or focus group segments, a process similar to code saturation. Also after coding, 2 team members analyzed the data to identify within- and across-group themes, patterns, and race and sex differences.

We developed a separate codebook for the interviews and coded these by using Microsoft Access 2003 (Microsoft Corporation, Redmond, Washington). Four research assistants listened to the audiotaped interviews. When a participant's response matched one of the codes, it was transcribed verbatim and coded in the database. This method of coding interviews was chosen because of the significant time and cost savings compared with traditional transcription. Coding was completed by 2 teams; each team coded the same interviews until reaching 80% intercoder agreement and then coded separately. After coding was complete, we counted and sorted the codes to identify major themes and patterns. Because of poor sound quality on some audiotapes, only 66 interviews (of 71 participants) were included in this analysis.

## Results

### Health concerns

During the focus groups, participants indicated that they most commonly worried about 1) chronic health conditions and 2) costs to treat these types of conditions. Chronic health conditions of most concern were obesity, diabetes, heart disease, hypertension, and high cholesterol. One participant said, "I worry about prostate cancer and diabetes . . . high blood pressure . . . hypertension." Another participant said, "Me personally, I think about heart disease is my concern." Participants worried most about conditions they or family members had, and shared stories about the difficulties of dealing with existing chronic health conditions. They also reported challenges in dealing with chronic conditions, including difficult interactions with medical professionals (eg, physicians who rush patients), particularly physicians' lack of time. Sex differences emerged — women were concerned about being overweight and obese whereas men worried more about health conditions that would inhibit their ability to work and provide money or health insurance for their families.

Across groups, respondents worried about the costs of health care associated with health conditions, including insurance, co-payments, and prescription costs. One male participant remarked, "Even those that can afford insurance can't afford to get sick." Some participants also worried about being unable to work because of a health issue. One man described his worry and the cascading effects of getting sick: "You get hurt on the job, the first thing they want to do is cut your insurance off, which they did to me. And then they start paying you $250 a week when you were used to making like $450 a week. And you get a lot of stress from that because if you're a single parent and you're raising kids and you're used to bringing home $500 a week, and they drop to $266 a week."

### Contributors to threat

Several factors contributed to feelings of severity and susceptibility for participants. Their own or family members' health experiences seemed influential in determining their personal susceptibility to health risks or conditions. Participants frequently mentioned they felt more likely to get conditions that "run in the family." As 1 participant said, "I mean, I've had high blood pressure and all that stuff. And it's not because I'm overweight . . . my mom had the same problem."

Race also emerged as a factor that influenced perceptions of risk or threat. Many African American participants were more concerned about diseases that disproportionately affected African Americans. Whereas, across groups, participants expressed concern about conditions such as high blood pressure, heart disease, cancer, stroke, and arthritis, many African American participants expressed the most concern about diseases that are most prevalent among African Americans: obesity, diabetes, hypertension, and heart disease.

### Decision making about health risks

When asked about barriers to health-protective behaviors, focus group participants commonly cited a lack of health insurance and conflicting information about how to protect themselves from health risks. Money was also a barrier, particularly for healthful eating. Specifically, participants stated that good-quality, healthful food was often too expensive to purchase. Additional barriers were work and family obligations, especially for female focus group participants. Many women said that taking care of their children and families kept them from being physically active. For example, 1 woman said, "My problem is trying to find a balance of work, school, momma, [and] health." Another participant cited time management as a barrier, suggesting that competing work and family demands thwarted her ability to consistently engage in health-protective behaviors.

In the individual interviews, apathy (lack of concern) was commonly reported as a barrier to protective behaviors, such as checking blood pressure, getting a seasonal influenza vaccination, and preparing for natural disasters. Men expressed much greater apathy about protecting themselves than did women and talked often about not being sick or never experiencing these risks. Many participants stated they did not check their blood pressure because they believed it was unimportant or because they were just lazy. As 1 woman noted, "I always make excuses: I don't have time, I'm fine, feel good, don't worry about it, not gonna happen to you." Natural disasters were slightly different; the lack of concern was often expressed in fatalistic statements such as "nothing can be done to prevent natural disasters" or that whatever happens is part of "God's plan." There was little motivation to prepare for emergencies, such as tornadoes, hurricanes, or other natural disasters.

Response efficacy concerns also emerged in interviews, especially regarding the influenza vaccine, physical activity, and seatbelt use. Participants expressed a great deal of concern about and distrust of the influenza vaccination, and some believed it would actually cause influenza instead of protecting them from it. A man recalled, "I'd never had the flu, and I said, 'let me take it as a precaution.' Once I took the flu shot, I caught the flu. . . . Next year I said 'no' [to the flu shot]." Participants also expressed concern about physical activity because of the potential for injury and pain. Seatbelts often were described as uncomfortable. White male participants most often described response efficacy concerns, followed by African American female participants.

### Health-protective behaviors

Despite efficacy concerns and other barriers, low-income focus group and interview participants reported engaging in health-protective behaviors. In the focus groups, participants commonly stated that eating healthful foods and being physically active were behaviors that could protect them from health conditions and diseases (especially chronic ones). One male participant said that "exercise, eating habits, of course, and then prevention care" would help protect his health. Some participants said they avoided using illegal drugs, did not drink alcohol, or drank only in moderation to protect their health. Others reported that taking medication regularly was an important health protective behavior, as was going to the doctor. One participant said he would "take my medication if I'm on medication, have the proper diet, and then exercise." Another participant said, "go to the doctor . . . get a physical every year."

In individual interviews, participants reported that the health-protective behaviors they most frequently engaged in were checking blood pressure and being physically active. The convenience of free machines in retail stores and the routine practice of blood pressure screening by health professionals were reasons given for frequent blood pressure checks. One woman noted that "Hypertension runs in my family, so I try to keep a check on [blood pressure]. I'll check it at Walmart when I'm at Walmart, which is a lot." Although participants reported that they were considerably physically active, they described the activity as being part of their job or home life (ie, walking their dog, cleaning their house, doing construction or landscaping work) rather than as being part of a typical exercise program (eg, running, walking, bicycling).

## Discussion

Our findings suggest that people living in poverty are aware of and concerned by the threats posed by chronic disease. This finding is important for this population because more poverty exists in the southern United States than in other parts of the country, and socioeconomic status remains one of the strongest determinants of health disparities (31-33). Our results suggest that family history, race, personal experience, and the absence of financial resources and insurance are reasons why some health risks are more threatening than others. Therefore, a health risk can seem threatening not only because of the severity of and susceptibility to the condition but also because of the financial and family implications that accompany it.

However, even when threat is high, several factors may prevent low-income Southerners from engaging in health-protective behaviors. First, the recommended protective responses may not seem effective. Low-income people may require greater reassurance about the effectiveness of these behaviors and more confidence in their abilities to perform them. This message should be given with sensitivity to the economic realities this population faces. Studies show time is a premium for the poor, and family responsibilities may limit time for protective behaviors and access to health care (34,35). Therefore, public health professionals should design interventions that are realistic, given these time constraints, and support low-income people as they attempt to overcome these barriers.

Although the results of our study show that a lack of income influences health decision making, subtle differences were found within sex and racial groups. These findings should be considered when designing interventions because studies consistently show higher rates of illness and death for racial and ethnic minorities, as well as for people with limited income. African Americans in our study believed that they were at greater risk for some diseases because of their race and that certain conditions were more prevalent in their community. Therefore, messages that target African Americans should address feelings of a shared history of poor health status and attempt to increase feelings of control, despite this shared history.

Although our results are not generalizable to the entire US population, they are valuable to the development of health promotion interventions aimed at eliminating health disparities, particularly for people living in poverty in the southern United States. When designing interventions to be used with this audience, researchers must recognize that many factors, not just perceived severity and susceptibility, determine whether health risks are overwhelming or threatening. Interventions developed for this audience must also specify how to address barriers to health-protective behaviors and concerns about the effectiveness of health-protective behaviors. Our results also highlight the importance of segmenting the audience and using different strategies across races and sexes, as well as the value of conducting comparative research with higher income groups when conducting focus groups.

During the past 2 decades, the nation's health has improved, but there remains a profound and disturbing variability in health status among different groups of people in the United States, particularly people from different social, economic, and cultural groups (36). Although social and cultural factors influence health, Renaud (37) argues that the biggest gap in health status is determined by whether one is rich or poor. Our results provide a deeper understanding of the health experiences and perceptions of health of people who live in poverty, a topic minimally researched and poorly understood.

## References

[1] Pamuk E, Makuc D, Heck K, Reuben C, Lochner K (1998). Socioeconomic status and health chartbook. Health, United States, 1998.

[2] Gaziano C, Horowitz AM (2001). Knowledge gap on cervical, colorectal cancer exists among US women. Newspaper Research Journal.

[3] Viswanath K, Breen N, Meissner H, Moser RP, Hesse B, Steele WR (2006). Cancer knowledge and disparities in the information age. J Health Commun.

[4] Lantz PM, House JS, Lepkowski JM, Williams DR, Mero RP, Chen J (1998). Socioeconomic factors, health behaviors, and mortality: results from a nationally representative prospective study of US adults. JAMA.

[5] Hiatt RA, Klabunde C, Breen N, Swan J, Ballard-Barbash R (2002). Cancer screening practices from national health interview surveys: past, present, and future. J Natl Cancer Inst.

[6] Wardle J, McCaffery K, Nadel M, Atkin W (2004). Socioeconomic differences in cancer screening participation: comparing cognitive and psychosocial explanations. Soc Sci Med.

[7] Pleis JR, Lucas JW (2009). Summary health statistics for U.S. adults: National Health Interview Survey, 2007. Vital Health Stat 10.

[8] Lochner K, Pamuk E, Makuc D, Kennedy BP, Kawachi I (2001). State-level income inequality and individual mortality risk: a prospective, multilevel study. Am J Public Health.

[9] Gorey KM, Vena JE (1995). The association of near poverty status with cancer incidence among black and white adults. J Community Health.

[10] Kaplan GA, Keil JE (1993). Socioeconomic factors and cardiovascular disease: a review of the literature. Circulation.

[11] Robbins JM, Vaccarino V, Zhang H, Kasl SV (2005). Socioeconomic status and diagnosed diabetes incidence. Diabetes Res Clin Pract.

[12] Singh GK, Miller BA, Hankey BF, Edwards BK (2003). Area socioeconomic variations in US cancer incidence, mortality, stage, treatment, and survival, 1975-1999 (NIH publication no. 03-0000).

[13] Halliday T, Taira DA, Davis J, Chan H (2007). Socioeconomic disparities in breast cancer screening in Hawaii. Prev Chronic Dis.

[14] Patterson RE, Haines PS, Popkin BM (1994). Health lifestyle patterns of US adults. Prev Med.

[15] US Census Bureau American FactFinder. 2006-2008 American Community Survey 3-year estimates, Columbia city, South Carolina. Poverty status in the past 12 months.

[16] US Census Bureau American FactFinder. 2006-2008 American Community Survey 3-year estimates, Birmingham city, Alabama. Poverty status in the past 12 months.

[17] US Census Bureau American FactFinder. 2006-2008 American Community Survey 3-year estimates, Jackson city, Mississippi. Poverty status in the past 12 months.

[18] US Census Bureau American FactFinder. 2006-2008 American Community Survey 3-year estimates. Percent of people below poverty level in the past 12 months (from whom poverty status is determined) 2008.

[19] US Census Bureau American FactFinder. 2007 American Community Survey 3-year estimates. Percent of people below poverty level in the past 12 months (from whom poverty status is determined) 2007.

[20] The Kaiser Family Foundation.

[21] Kung HC, Hoyert DL, Xu J, Murphy SL (2008). Deaths: final data for 2005. Natl Vital Stat Rep.

[22] Heron M, Hoyert DL, Murphy SL, Xu J, Kochanek KD, Tejada-Vera B (2009). Deaths: final data for 2006. Natl Vital Stat Rep.

[23] Prevalence and trends data, tobacco use — 2008. Adults who are current smokers.

[24] (2008). Prevalence and trends data overweight and obesity — 2008. Weight classification by body mass index (BMI).

[25] (2008). Prevalence and trends data diabetes — 2008. Have you ever been told by a doctor that you have diabetes?.

[26] DeNavas-Walt C, Proctor BD, Smith J (2007). Income, poverty, and health insurance coverage in the United States: 2006 (contract no. P60-233).

[27] Witte K (1998). Theory-based interventions and evaluations of outreach efforts.

[28] Krueger RA (1994). Focus groups: a practical guide for applied research.

[29] Calderón JL, Baker RS, Wolf KE (2000). Focus groups: a qualitative method complementing quantitative research for studying culturally diverse groups. Educ Health (Abingdon).

[30] Glaser BG, Strauss A (1967). A discovery of grounded theory: strategies for qualitative research.

[31] Adler NE, Boyce WT, Chesney MA, Folkman S, Syme SL (1993). Socioeconomic inequalities in health. No easy solution. JAMA.

[32] Williams DR, Collins C (2001). Racial residential segregation: a fundamental cause of racial disparities in health. Public Health Rep.

[33] Holt JB (2007). The topography of poverty in the United States: a spatial analysis using county-level data from the community health status indicators project. Prev Chronic Dis.

[34] Romeo JH (2005). Down and out in New York City: a participant-observation study of the poor and marginalized. J Cult Divers.

[35] Eyler AA, Matson-Koffman D, Vest JR, Evenson KR, Sanderson B, Thompson JL (2002). Environmental, policy, and cultural factors related to physical activity in a diverse sample of women: The Women's Cardiovascular Health Network Project — introduction and methodology. Women Health.

[36] (2002). National Institutes of Health. Strategic research plan and budget to reduce and ultimately eliminate health disparities.

[37] Renaud M, Evans G, Barer ML, Marmor TR (1994). The future: Hygea versus Panakeia. Why are some people healthy and others are not? The determinants of health of populations.

